# A low-noise silicon nitride nanopore device on a polymer substrate

**DOI:** 10.1371/journal.pone.0200831

**Published:** 2018-07-20

**Authors:** Wook Choi, Eun-Seok Jeon, Kyoung-Yong Chun, Young-Rok Kim, Kyeong-Beom Park, Ki-Bum Kim, Chang-Soo Han

**Affiliations:** 1 School of Mechanical Engineering, College of Engineering, Korea University, Anam-Dong, Seongbuk-Gu, Seoul, Korea; 2 Institute of Life Sciences and Resources and Department of Food Science and Biotechnology, College of Life Sciences, Kyung Hee University, Yongin, Korea; 3 Department of Materials Science and Engineering, Seoul National University, Seoul, Korea; Institute of Materials Science, GERMANY

## Abstract

We report a novel low-noise nanopore device employing a polymer substrate. The Si substrate of a fabricated Si-substrate-based silicon nitride (Si_3_N_4_) membrane was replaced with a polymer substrate. As such, laser machining was used to make a micro-size hole through the polyimide (PI) substrate, and a thin Si_3_N_4_ membrane was then transferred onto the PI substrate. Finally, a nanopore was formed in the membrane using a transmission electron microscope for detection of biomolecules. Compared to the Si-substrate-based device, the dielectric noise was greatly reduced and the root-mean-square noise level was decreased from 146.7 to 5.4 pA. Using this device, the translocation of double-strand deoxyribonucleic acid (DNA) was detected with a high signal/noise (S/N) ratio. This type of device is anticipated to be available for future versatile sequencing technologies.

## Introduction

Nanopore devices are considered excellent tools for the sensing and characterization of several biomolecules such as deoxyribonucleic/ribonucleic acid (DNA/RNA), proteins, and DNA-protein complexes at the single molecule level [1–8]. Nanopore systems can be broadly classified into biological nanopores and solid-state nanopores. Biological nanopores are generally based on α-hemolysin [9–11] or MspA [12–15], which have excellent sensitivity with a very low noise level; however, the measurement conditions are limited due to the fragile lipid bilayer and fixed pore diameter of these species [12,16]. Recent studies have reported improvements in the performance of biological nanopore systems such as phi29 polymerase enzymes [15,17]. However, solid-state-based sensing platforms have also attracted increasing attention as alternatives to biological nanopore systems due to the well-established manufacturing processes for the former. Since the first reports of the fabrication of silicon nitride nanopores using an argon ion beam [18], techniques for the fabrication of solid-state nanopore devices have been developed using a variety of materials such as silicon nitride [19], silicon oxide [20], graphene [21], boron nitride [22], hafnium oxide [23], and molybdenum disulfide [24]. In general, insulating materials are favored as the nanopore membrane for solid-state nanopore devices because they are highly stable in harsh chemical solutions such as sulfuric acid and at high temperatures. In particular, films of silicon nitride and silicon oxide have advantages such as robust and controllable thickness and pore size. Further, their surfaces are readily chemically modified, and the semiconducting processes are well-established. These solid-state nanopore devices exhibit the potential to detect many types of molecules [7,18,21,25–29]. However, this type of device shows a significantly higher background noise than those based on biological nanopores in ionic current measurements [30]. In general, solid-state nanopores consist of a silicon nitride membrane on a silicon substrate and have a background noise level of several hundred pA. On the other hand, the typical noise level of biological nanopores composed of lipid bilayers is typically sub-10 pA RMS.

Several attempts have been made to effectively reduce the dielectric noise, such as by depositing a thick dielectric layer under the Si_3_N_4_ membranes [19,31,32], coating with polydimethylsiloxane (PDMS) [32–34], the use of glass nanopores [35–37], and modification of substrate materials such as Pyrex [38,39] or quartz [40]. All of these methods result in significantly reduced background noise levels, but they require semiconducting or tricky manufacturing process to form micro or sub-micron holes in the substrate that supports the nanopore membrane. Simple and reliable fabrication steps are desirable for the fabrication process because complex and tricky processes may lead to a low yield and poor device quality [41]. Solid materials such as SiO_2_ and Pyrex have been used as the supporting material for the nanopore membrane, but this makes the device fragile and tricky to handle. In order to avoid the fragility issue and improve the noise characteristics, polymeric materials have been considered. In a previous study, a polymeric nanopore membrane fabricated by a track etching technique was used for biomolecule sensing, where semiconducting processing technology was not required [42]. However, this technique produces many pores with a large pore size, and a relatively thick film, features that are not desirable for solid-state nanopore devices and DNA detection [43,44]. Therefore, polymeric materials such as polyimide (PI) and polyethylene terephthalate (PET) are more suitable as supporting materials than nanopore membranes. In particular, PI exhibits excellent mechanical properties, thermal stability, chemical resistance, and a high breakdown voltage. In addition, the high dielectric constant of PI is useful for reducing the current noise generated by the device capacitance [44–46]. To circumvent the limitations of the track etching technique [47], laser ablation is employed herein because the required pore size of PI is about a micrometer or more. As the nanopore membrane, Si_3_N_4_ that can be reliably fabricated by a well-established process is employed. Silicon nitride (SiN) films are widely used in micro-electromechanical systems (MEMS), such as integrated circuits and photonic applications, and for surface passivation, layer insulation, and in dielectric capacitors due to their excellent physical and chemical properties, high density and dielectric constant, and good insulating properties and excellent biocompatibility [48–50]. Therefore, we could achieve a low-noise nanopore device based on a relatively simple fabrication method. Therefore, the use of low-noise materials and relatively easy manufacturing methods provide a promising path for improving the signal-to-noise ratios to facilitate electrical identification of a single molecule.

In this study, we demonstrate the fast and simple fabrication of a micro-hole substrate via laser drilling on a PI substrate. After placing a Si_3_N_4_ membrane on top of the polymer substrate, the nanopores are sculpted on the Si_3_N_4_ membrane using transmission electron microscopy (TEM). The noise of the ionic current achieved with the PI, Si, and Pyrex substrate-based Si_3_N_4_ nanopore membrane is analyzed, demonstrating that the noise level of the PI substrate is much lower than that of the Si substrate, but comparable to that of the Pyrex substrate. Double-strand DNA is successfully translocated using the PI substrate-based Si_3_N_4_ nanopore device with a high signal-to-noise ratio.

## Experimental results

**Fig 1:**
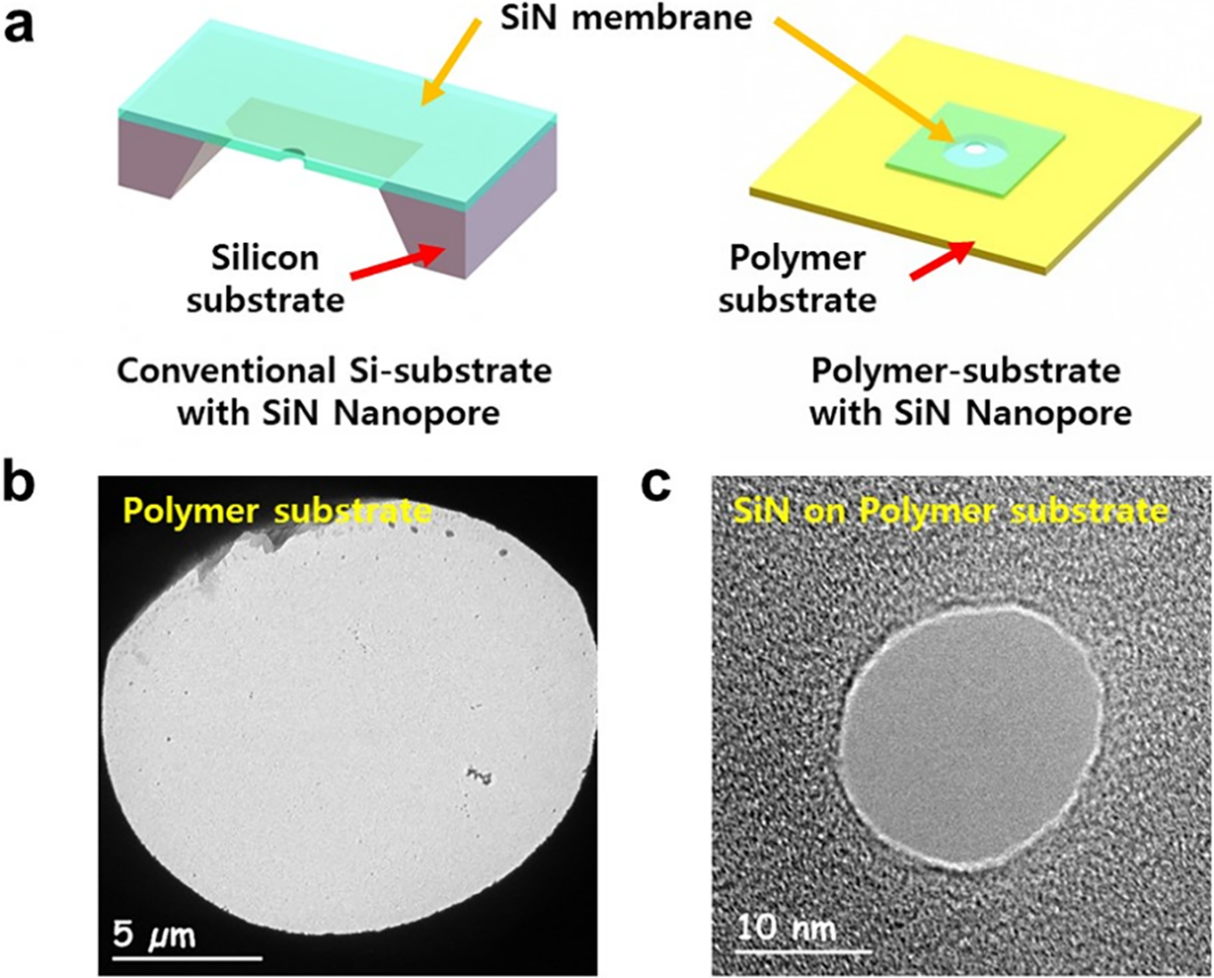
Polymer substrate-based nanopore device. **(a)** Schematic illustrations of polymer-substrate-based nanopore device consisting of a micro-meter sized pore in a polymer substrate and 20 nm thick free-standing SiN membrane. TEM images of **(b)** micro-meter size pore drilled in polymer substrate by laser ablation and **(c)** nanopore with 8 nm diameter drilled by a highly focused electron beam using TEM.

Fig 1A presets a schematic diagram of the conventional Si substrate-based Si_3_N_4_ nanopore vs. the polymer substrate-based Si_3_N_4_ nanopore device. It might be difficult to directly compare the steps used in processing the Si and PI substrates because fabrication of the Si_3_N_4_ thin membrane requires several semiconducting processes. Nevertheless, the current approach shows the potential for reducing the process steps significantly by the transfer of other nano-membranes onto the substrate. In this study, we used a polymer-based nanopore device employing the PI substrate to achieve low dielectric noise as well as facile fabrication. For this, a hole was drilled through the flexible polymer substrate using a laser ablation system, and the Si_3_N_4_ membrane was transferred onto it as an example of a nano-membrane. This PI film exhibits very high stability in the electrolyte solution and high machinability for laser milling. Moreover, this material presents low dielectric noise due to the insulating nature. Fig 1B and 1C show the hole through the PI film and the nanopore through the Si_3_N_4_ membrane, respectively.

**Fig 2:**
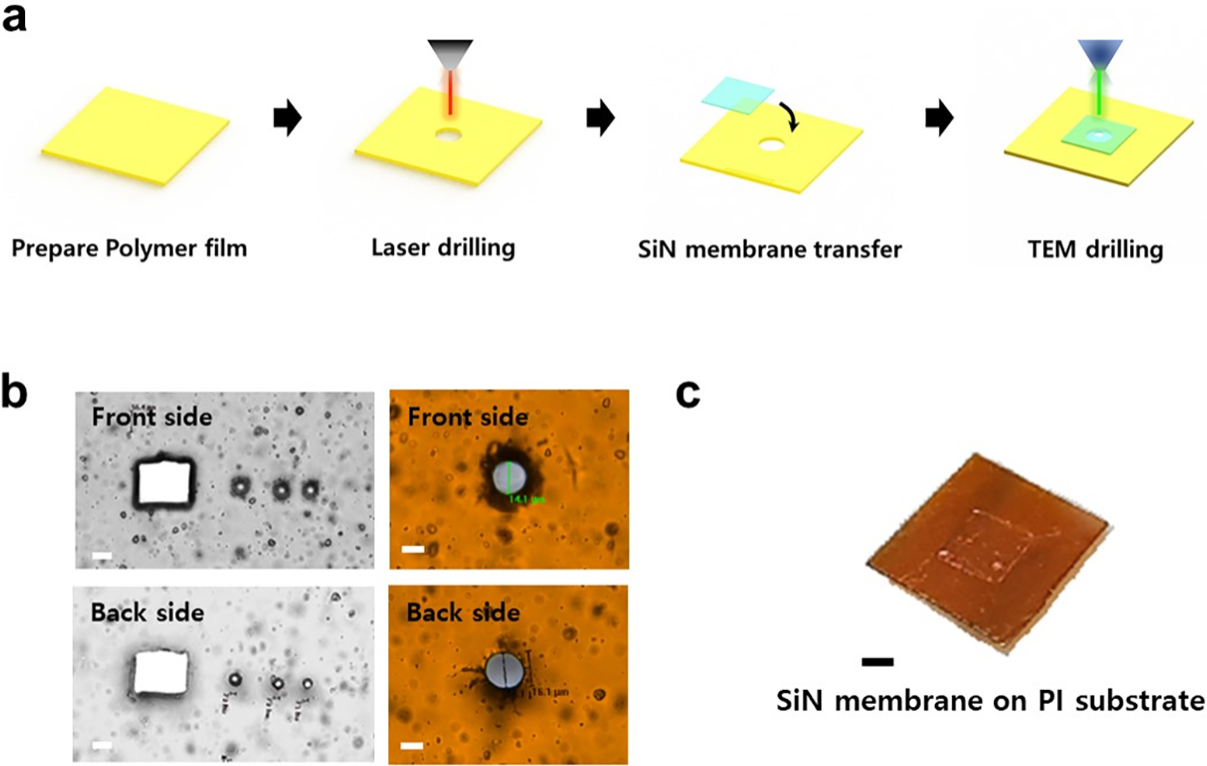
Process for fabrication of the PI substrate-based Si_3_N_4_ nanopore device. **(a)** Preparation of polymer film with 75 μm thickness, micro-size hole drilled by laser ablation, transfer of SiN membrane grown by LPCVD onto perforated polymer substrate, and fabrication of nanopore on the suspended SiN membrane using TEM. **(b)** Optical micrograph of micro-size holes of various diameters and shapes in polymer substrate, formed by laser ablation. The top photo shows the front side and the bottom photo shows the back side. The scale bar is 5 μm. **(c)** Photograph of SiN membrane on PI substrate. The scale bar is 1 mm.

The procedure for fabrication of the polymer substrate-based nanopore is shown in Fig 2A. Firstly, a 75 μm thick polymer-based substrate was cut into dimensions of 5 × 5 cm^2^, and then a sub-15 μm hole was drilled using a Laser Microdissection (LMD6, Leica, Wetzlar, Germany) system. A 20 nm thick Si_3_N_4_ membrane was grown by low pressure chemical vapor deposition (LPCVD) and the thickness was controlled by reactive ion etching (RIE); the membrane was transferred onto a perforated polymer (PI) substrate. The transfer method is the so-called “fishing method”, which is similar to the conventional method of transferring graphene or other 2D materials, and is similar to the method of transferring a Si_3_N_4_ membrane to a Pyrex substrate [38,39]. After transfer of the Si_3_N_4_ membrane onto a polymer substrate with a 15 μm opening, nanometer-sized pores were drilled into the Si_3_N_4_ membrane using TEM (Tecnai G2 F30, FEI Co.) with the sample holder, as illustrated in Fig 1C. Fig 2B illustrates the results of fabricating the various holes on the PI film using laser ablation. The size and shape of the hole could be controlled, but the feasible size of the hole is limited due to the laser power and wavelength. In this experiment, the thickness of the PI film is critical for determining the minimum size of the hole. According to previous studies, the noise level is closely related to the surface coverage and the dielectric constant of the passivating materials [33,34]. Therefore, the pore size was optimized to minimize the area exposed to the electrolyte, and we considered 25, 50, and 75 μm PI films as candidate substrates. Considering the laser process and substrate performance, the 75 μm thick PI film was selected because the thinner film was distorted in the TEM chamber due to the vacuum pressure. Nevertheless, we could drill a circular hole with a diameter of 15 μm. As shown in Fig 2B, the shape of the hole was almost identical on the back-side and front-side of PI, with a near cylindrical shape. The thin Si_3_N_4_ membrane was very fragile, requiring caution in the transfer process. Using an optical microscope, we adjusted the position of the Si device and manually fixed it on the device on the PI substrate (Fig 2A). When the Si device was detached from the PI film, the Si_3_N_4_ thin membrane remained on the PI film. Fig 2C shows the Si_3_N_4_ membrane attached to the PI film. The membrane could be successfully transferred onto the PI film within a minute.

### Ionic current measurements for the nanopore device

To measure the ionic current of the PI substrate-based Si_3_N_4_ nanopore device, the sample was treated with oxygen plasma (Covance, Femto Science, Korea) for 1 min at 70 W to enhance the wettability of the sample. The nanopore chip was then mounted on a custom-made flow cell with polydimethylsiloxane (PDMS) gaskets and chambers (Cis- and Trans-), where the cell was filled with 1 M KCl solution containing TE buffer (10 mM Tris-HCl and 1 mM EDTA at pH 8.0). The Ag/AgCl electrodes were inserted into both chambers and then connected to an Axopatch 200B amplifier (Axopatch 200B, Axon Instrument Co.) with recording at a sampling rate of 250 kHz with low-pass filtering (4-pole Bessel filter) at 10 kHz. The data were acquired and analyzed using a patch clamp and Clampfit software, respectively. All measuring wires and the fluid cell were placed in a Faraday cage for shielding and grounding to avoid unwanted noise.

**Fig 3:**
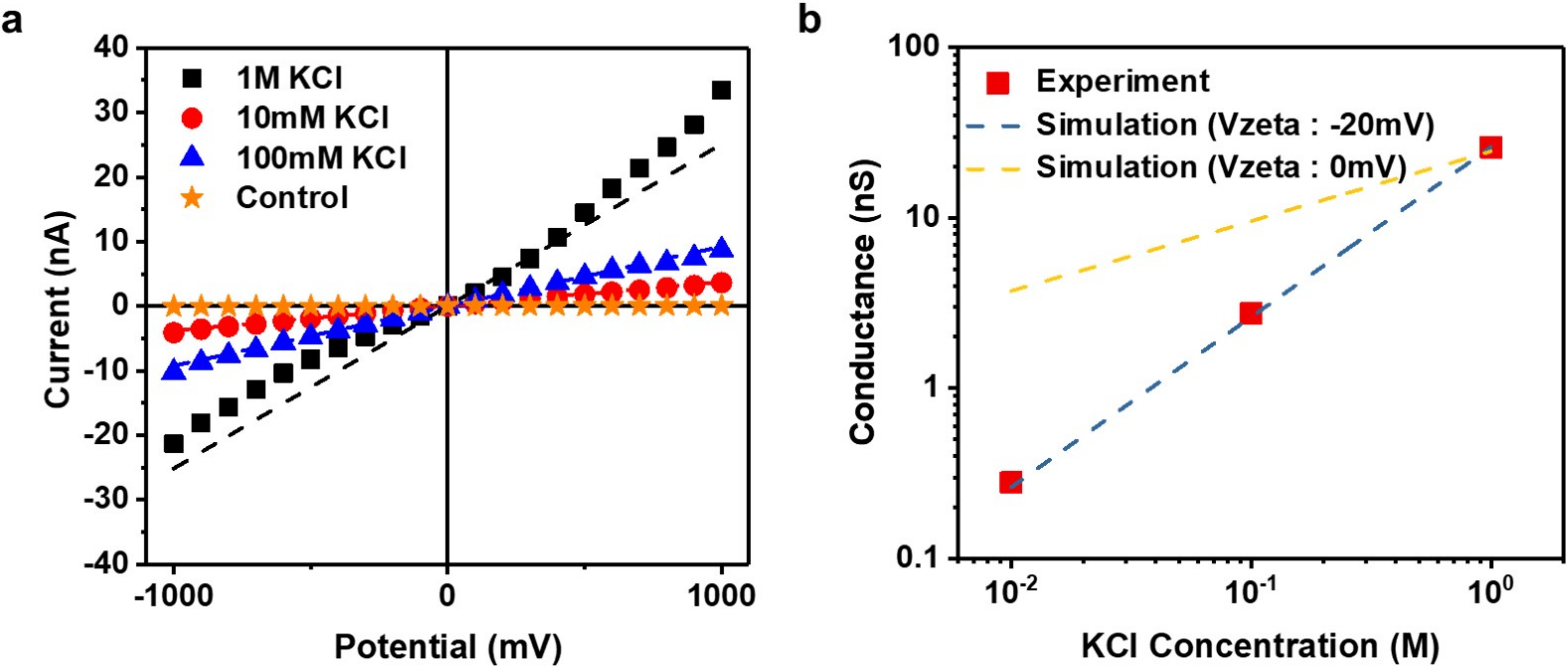
Comparison of experimental and simulated conductance for various electrolyte concentrations. **(a)** *I-V* characteristics of polymer-based nanopores, measured with various concentrations of potassium chloride electrolyte. **(b)** Experimental (red square) and simulated conductance (blue and yellow dotted line) of the polymer-based nanopore device measured at various concentrations of potassium chloride electrolyte.

As shown in Fig 3A, the current-voltage (*I-V*) characteristics of the PI substrate-based 8-nm nanopore device were evaluated as a function of the KCl concentration. First, in order to confirm perfect adhesion between the Si_3_N_4_ membrane and PI substrate, a control experiment was performed using a Si_3_N_4_ membrane without nanopores. No current change was observed in the voltage range of −1 V to + 1 V, which means that there was no leakage between the SiN membrane and the PI substrate. The *I-V* curve showed linear ohmic behavior in the voltage range of −1 V to +1 V. The ionic conductivity can be calculated from the slope of the linear plot and is influenced by the surface charge density, number density of ions, ion mobility, and pore geometry. Therefore, as the concentration of potassium chloride increases (or decreases), the number density of ions increases (or decreases), increasing (or decreasing) the ionic conductivity, *G*. Herein, a clear decrease was observed with decreasing potassium chloride concentration. From the *I-V* measurement, the ionic conductance of the nanopore was 25.1 nS at 1 M KCl. The data from the theoretical calculation were compared with the experimental results. The size of the nanopore, measured by TEM, was 8 nm and the thickness of the Si_3_N_4_ membrane was 20 nm. The theoretical equations relating the ionic conductance to the diameter can be expressed as:

$$G_{nanopore}=\sigma_{KCl}{\left(\frac{{4h}_{eff}}{{\pi d}^{2}}+\frac{1}{d}\right)}^{-1}$$ (1)

where *σ_KCl_* (11.1 S m^-1^) is the molar conductivity of the electrolyte at 20°C, *h_eff_* is the effective thickness of the nanopore membrane, and *d* is the diameter of the nanopore obtained by TEM observation [51,52]. The theoretical nanopore size was 7.58 nm, where the experimental and theoretical values were in good agreement, but the slight difference seems to be due to the hourglass shape of the nanopores caused by the TEM drilling process. When the high-intensity TEM electron beam impinges on the silicon nitride membrane, sputtering of the Si and N atoms can occur on both sides of the membrane. This sputtering results in the formation of nanopores. Due to the intensity distribution around the center point, sputtering causes tilts on both sides of the membrane. Consequently, as the sputtering continues, an ‘hour-glass’ shape nanopore is created. The theoretical calculation assumes that the pores are cylindrical with no angles. However, the actual nanopore has a slight angle, and for this reason, the measured ionic conductance differs slightly from the theoretical expectation [53,54].

As shown in Fig 3B, computer simulations were conducted using COMSOL to verify that the experimental results fit well with the theoretical calculations (see Supporting information S1 Text). The conductance was modeled using the following equation:

$$G=\frac{(-J_{K,z}+J_{Cl,z})F\pi {r_{pore}}^{2}}{V_{in}-V_{out}}$$ (2)

This equation combines the Poisson-Boltzmann [55], Nernst-Planck [56], and Navier-Stokes equations for calculating the conductance from the electrical potential [57].

Here, *J_K,z_* and *J_Cl,z_* represent the ion flux in the Z-direction of the potassium and chlorine ions, respectively; F represents the Faraday constant (96,500 C mol^-1^); *r_pore_*, *V_in_*, and *V_out_* represent the pore radius, positive bias, and negative bias, respectively. The detailed simulation procedure is presented in the Supporting Information S2 Text. The simulations were performed under the same experimental conditions, i.e., bias voltage, electrolyte concentration, and surface charge of the Si_3_N_4_ membrane. Overall, the experimental value and the theoretical value were well matched when the Si_3_N_4_ membrane surface zeta potential was −20 mV.

### Noise analysis of the nanopore device

**Fig 4:**
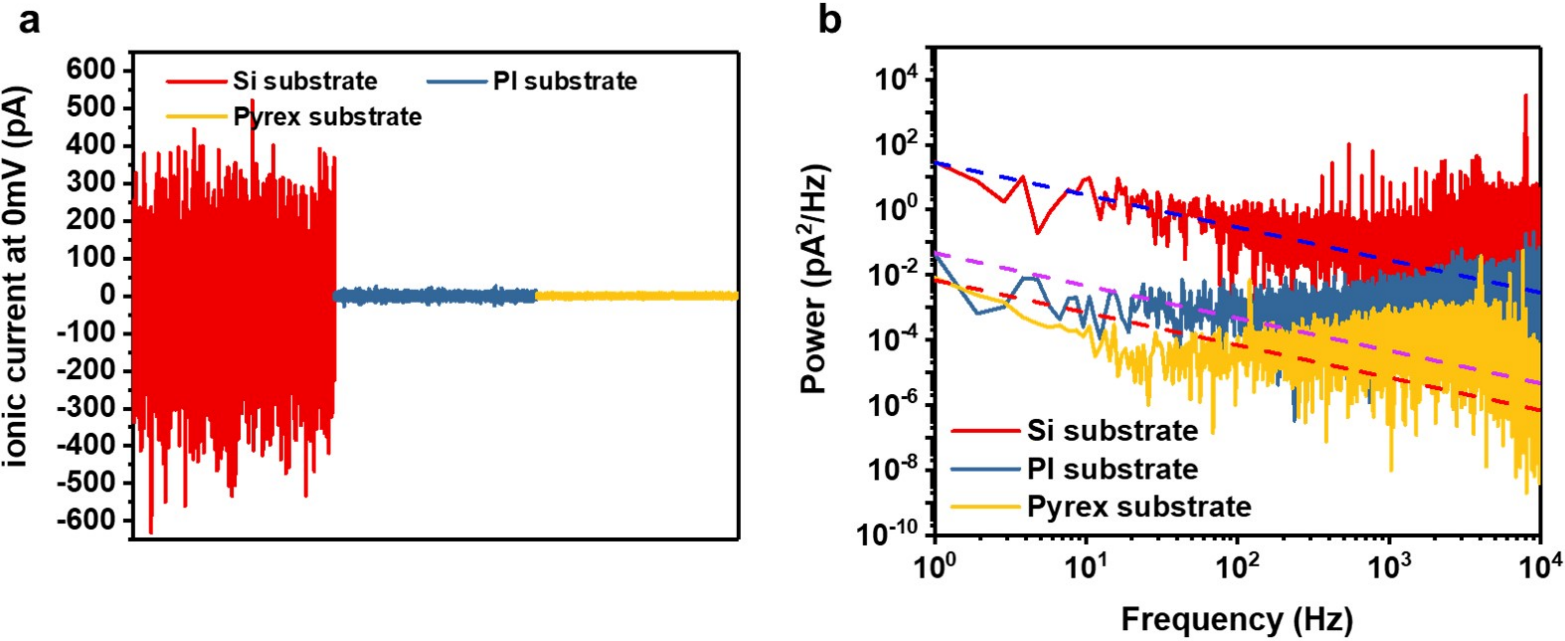
Ionic current traces and current power spectral densities with various substrates. **(a)** Baseline ionic current traces as a function of time for Si substrate (red), PI substrate (blue), and Pyrex substrate (yellow). **(b)** Measured power spectral density (PSD) of various substrates. The dashed fitted line represents flicker noise (1/*f*) of various substrates. All experiments were performed with 1 M KCl at 0 mV with a low-pass filter at 10 kHz.

Fig 4A shows the baseline ionic current trace for the three nanopore devices, measured at a sampling rate of 250 kHz using a low-pass 10 kHz filter. In order to compare the noise, we fabricated a Pyrex substrate-based device with a Si_3_N_4_ membrane, which was developed by another research group [39]. Si_3_N_4_ membranes with the same thickness were placed on top of Si, Pyrex, and PI substrates, respectively. The Pyrex substrate-based device and polymer substrate-based device respectively showed a peak noise of 12 pA (Irms = 2.6 pA) and 20 pA (Irms = 5.4 pA), which are significantly smaller than that (700 pA; Irms = 146.7 nA) observed for the Si substrate-based device. This result demonstrates that the noise level of the developed polymer substrate-based nanopore device is comparable to that of the Pyrex substrate-based device. In order to analyze the effect of the substrate material on the noise level, the same Si_3_N_4_ membrane was transferred to the Si substrate, Pyrex substrate, and PI substrate. The power spectral density curves measured at 0 mV (Fig 4B) clearly show that the PI- and Pyrex-substrate devices exhibited significantly reduced noise relative to the Si-substrate device over the entire frequency range. To investigate the noise characteristics in detail, we employed the noise spectrum expressed by the polynomial function, S = *Af*^−1^ + B + *Cf* + *Df*^2^, where *f* is the frequency in Hz. The parameters A, B, C, and D indicate flicker noise, Johnson and shot noise, dielectric noise, and electric noise, respectively [58].

The PI substrate and Pyrex substrate exhibited much lower values for all noise parameters than the Si substrate, as shown in Table 1. Notably, there was a dramatic change in parameter C, which is the dielectric noise. The dielectric noise determines the high-frequency range of the device noise, which can be reduced by minimizing the substrate capacitance. Therefore, this can be reduced by using highly insulating dielectric materials such as Pyrex or quartz, but an additional photolithography process is required. With simple fabrication, we achieved a low-noise nanopore device with a high dielectric constant by using the PI substrate.

**Table 1:** Comparison of the noise parameters of Si-, Pyrex-, and PI-substrate nanopore devices.

Noise parameters	Si substrate	Pyrex Substrate	PI substrate
**A**	2.71E+01	6.11E-03	3.77E-02
**B**	2.78E-02	1.14E-04	9.12E-04
**C**	1.57E-04	5.89E-08	8.13E-07
**D**	4.34E-09	-8.02E-12	-4.25E-11

### DNA translocation in the PI substrate-based Si_3_N_4_ nanopore device

**Fig 5:**
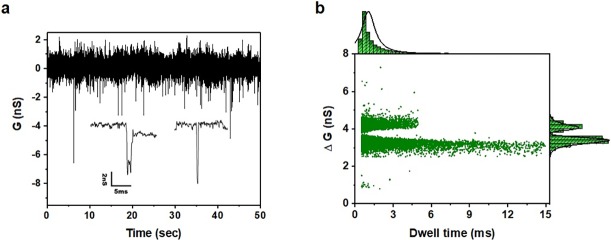
DNA translocation through PI substrate-based Si_3_N_4_ nanopore device. **(a)** Ionic conductance trace as function of time with passage of λ-DNA through polymer-substrate-based SiN nanopore. **(b)** Conductance blockage versus translocation event duration scatter diagram with histogram of λ-DNA translocation at 200 mV for polymer-based nanopore. A Gaussian distribution fit was performed to characterize the average dwell time and conductance blockade.

To conduct the DNA translocation experiment, the nanopore device was initially treated with oxygen plasma to enhance the wettability. The PI substrate-based SiN nanopore device was then mounted on a flow cell and filled with 1 M KCl containing TE buffer solution, as described in the section on measurement of the ionic conductance. The double-stranded DNA sample (48.5 kbps λ-DNA, Bioneer Co.) was diluted to 1 nM and inserted into the Cis chamber after stabilization of the ionic current. The Ag/AgCl electrodes in both chambers supplied the bias voltage, and the computer was used to monitor the change in the ionic current and DNA translocation in real time with passage from the Cis to the Trans chamber. Fig 5A shows the change in the ionic conductance due to DNA translocation as a function of time. The signal-to-noise ratio of the translocation signal appeared to be very high, but there was a slight fluctuation of the amplitude. Further, as illustrated in the bottom inset in Fig 5A, two types of translocation events that differ in terms of the ionic conductance and dwell time were detected. These events occur when the DNA molecules traverse the nanopore, occupy part of the nanopore, and block the current flow. In the present device, the DNA molecules passing through the nanopore are translocated in a linear or folded form; thus, the change in the DNA dwell time and ionic conductance show different features. To investigate the DNA translocation events in the PI substrate-based Si_3_N4 nanopore device, we present the marginal histogram of the events for λ-DNA interacting with the 8 nm nanopore at +200 mV bias voltage in Fig 5B. Each of the DNA translocation events is represented as a separate point on this scatter plot. Here, we can clearly see two populations along the *ΔG* axis. This represents different dwell times and ionic conductance values depending on the linear and folded forms in which the DNA molecules pass through the nanopore, as described earlier. The right panel in Fig 5B shows the histogram of the ionic conductance blockade fitted with a double Gaussian peak. The first peak (upper) shows the linear translocation of a single DNA strand through the nanopore with *ΔG* = 3.4 nS, whereas the second peak (lower) in the histogram corresponds to the translocation of folded DNA through the nanopore with *ΔG* = 4.1 nS. In addition, the upper panel in Fig 5B also shows a single Gaussian peak for the translocation time, with the center value of 1.1 msec. These DNA translocation times correspond very well to that reported for silicon nitride and quartz nanopores [20,37,59].

## Conclusions

In summary, we proposed a low-noise Si_3_N_4_ nanopore device developed by transfer to a polymer substrate. The 15 μm thru-hole of the PI film was formed by laser ablation, the 20 nm Si_3_N_4_ membrane on the Si substrate was manually transferred onto the PI substrate, and the nanopore was sculpted on the membrane using the TEM instrument. Notably, the features of the PI film were well maintained during TEM milling. The measured ionic conductance through the nanopore was well matched with the theoretical calculation. In addition, noise analysis data for the three types of nanopore devices demonstrate that the developed PI-based nanopore device exhibits significant noise reduction over a wide frequency range, making it superior to the Si substrate-based device. This polymer substrate-based nanopore device tackles the technical hurdles of high noise and complex manufacturing. Thus, this approach will be helpful for accelerating technological advancement in the biomedical sensing field.

## Supporting information

S1 Text Equation for the nanopore conductance. (DOCX)

S2 Text Simulation using COMSOL. (DOCX)
