# Supplementary material for: A low-noise silicon nitride nanopore device on a polymer substrate
Source: PLoS One. 2018 Jul 20;13(7):e0200831. doi: 10.1371/journal.pone.0200831 (PMC6054398; doi:10.1371/journal.pone.0200831)
Supplement: S1 Text — (DOCX) [file pone.0200831.s001.docx]

**S1 Text. Equation for the nanopore conductance:**

The governing equations are typically composed of three equations: the Poisson-Boltzmann Equation (PBE), the Nernst-Planck Equation (NPE), and the Navier-Stokes Equation (NSE). PBE was used to define the electric double layer phenomenon acting on the walls of the electrolyte-filled nanopore and also used to define the electric filed in the nanopore system. NPE calculates the behavior of the electrolyte by diffusion, electric field, and flow. NSE was used to calculate the ion flux of an electrolyte caused by the electro-osmosis flow. First, using PBE, we can derive an equation that can obtain surface charge density and electric potential, and the equation can be expressed as:

$\sigma_{0}=\varepsilon\kappa\Phi_{0}, \kappa^{-1}=\left[ \frac{\varepsilon_{0}DR^{2}T}{2F^{2}Z^{2}k_{B}c_{bulk}} \right]^{\frac{1}{2}}$ (1)

Here, $\sigma_{0}$ is the surface charge density, $\varepsilon$ is relative permittivity, $\Phi$ is electrical potential, $\kappa^{-1}$ is Debye length, $k_{B}$ is Boltzmann constant, $D$ is diffusion coefficient, $c$ is concentration of ions, $Z$ is number of electrons, $F$ is Faraday constant, $R$ is ideal gas constant , and $T$ is temperature.

The ion movements in an electrolyte can be represented by a single equation that combines NSE and NPE:

$\vec{J}=\vec{J}_{diff}+\vec{J}_{elec}+\vec{J}_{flow}$ (2)

$$\vec{J}_{diff}=-D\nabla c, \vec{J}_{elec}=\frac{zFDc}{RT}\nabla\Phi, \vec{J}_{flow}=c\vec{v}_{flow}$$

$\vec{J}=-D\nabla c+\frac{zFDc}{RT}\nabla\Phi+c\vec{v}_{flow}$ (3)

Here, $\vec{J}$ is total ion flux, $\vec{J}_{diff}$ ion flux by diffusion, $\vec{J}_{elec}$ ion flux by electric field, and $\vec{J}_{flow}$ ion flux by flow. Also, $D$ is the diffusion coefficient, $c$ is the concentration of ions, *z* is the number of electrons, *F* is the Faraday constant, *R* is the ideal gas constant, *T* is temperature, and $\Phi$ is the electric potential.

The ionic current can be derived by multiplying the ion flux and the Faraday constant and the number of electrons obtained, and is expressed as:

$I=F\sum_{i} z_{i}\vec{J}_{i}$ (4)

Where, $I$ is ionic current, $z$ the number of electrons, and $F$ the Faraday constant.

Conductance G are defined as ionic current divided by potential difference, which can be finally summarized as:

$G=\frac{(-J_{K,z}+J_{Cl,z})F\pi{r_{pore}}^{2}}{V_{in}-V_{out}}$ (5)

**S2 Text.** **Simulation using COMSOL**

***Properties***

1. Initial Value
   - Temperature (T): 293.15 [K]
   - Pressure (Pa): 1 [atm]
   - Electric potential: 0 [V]
   - Ion Concentration (C): 10, 100, 1000 [mol/m3]
   - Velocity field: 0 [m/s]
2. Properties of Materials
   - Charge number of potassium ion (Z_K): 1
   - Charge number of chloride ion (Z_Cl): -1
   - Diffusion coefficient of potassium ion (D_K): 1.957e-9 [m2/s]
   - Diffusion coefficient of chloride ion (D_Cl): 2.032e-9 [m2/s]
   - Electrophoretic mobility of potassium ion (mu_K): 7.7469e-8 [m2/(V·s)]
   - Electrophoretic mobility of chloride ion (mu_Cl): 8.0438e-8 [m2/(V·s)]
   - Density of KCl solution (rho_KCl): 1000 [kg/m3]
   - Viscosity of KCl solution (nu_P): 1.002e-3 [Pa*s]
   - Relative permittivity of KCl solution (e): 80
3. Constants
   - Faraday constant (F): 96500 [C/mol]
   - Ideal gas constant (R): 8.314 [J/(mol·K)]
   - Boltzmann constant **(k)**: 1.3806 [m^2^·kg/(s^2^·K)]
4. Dimensions of Model
   - Radius of reservoir **(r_res)**: 3 [um]
   - Radius of nanopore **(r_pore)**: 4 [nm]
   - Length of nanopore **(l_pore)**: 20 [nm]

**1**

**2**

**3**

**4**

**Boundary Conditions**

1. Electrostatics: Poisson-Boltzmann Equation
   [#1] Positive bias **(V_in)**: 200 [mV]
   [#2] Negative bias **(V_out)**: 0 [mV]
   [#4] Surface charge density **(sc_mem)**: 0, -20 [mC/m^2^]
2. Transport of Diluted Species: Nernst-Planck Equation
   [#1~#4] No flux: $-n\cdot N=0$
3. Laminar Flow: Navier-Stokes Equation
   [#1~#2] Outlet: 0 [Pa]
   [#3~#4] Wall: u = 0
