# Supplementary material for: A low-noise silicon nitride nanopore device on a polymer substrate
Source: PLoS One. 2018 Jul 20;13(7):e0200831. doi: 10.1371/journal.pone.0200831 (PMC6054398; doi:10.1371/journal.pone.0200831)
Supplement: S2 Text — (DOCX) [file pone.0200831.s002.docx]

**S2 Text.** **Simulation using COMSOL**

***Properties***

1. Initial Value
   - Temperature (T): 293.15 [K]
   - Pressure (Pa): 1 [atm]
   - Electric potential: 0 [V]
   - Ion Concentration (C): 10, 100, 1000 [mol/m3]
   - Velocity field: 0 [m/s]
2. Properties of Materials
   - Charge number of potassium ion (Z_K): 1
   - Charge number of chloride ion (Z_Cl): -1
   - Diffusion coefficient of potassium ion (D_K): 1.957e-9 [m2/s]
   - Diffusion coefficient of chloride ion (D_Cl): 2.032e-9 [m2/s]
   - Electrophoretic mobility of potassium ion (mu_K): 7.7469e-8 [m2/(V·s)]
   - Electrophoretic mobility of chloride ion (mu_Cl): 8.0438e-8 [m2/(V·s)]
   - Density of KCl solution (rho_KCl): 1000 [kg/m3]
   - Viscosity of KCl solution (nu_P): 1.002e-3 [Pa*s]
   - Relative permittivity of KCl solution (e): 80
3. Constants
   - Faraday constant (F): 96500 [C/mol]
   - Ideal gas constant (R): 8.314 [J/(mol·K)]
   - Boltzmann constant **(k)**: 1.3806 [m^2^·kg/(s^2^·K)]
4. Dimensions of Model
   - Radius of reservoir **(r_res)**: 3 [um]
   - Radius of nanopore **(r_pore)**: 4 [nm]
   - Length of nanopore **(l_pore)**: 20 [nm]

**1**

**2**

**3**

**4**

**Boundary Conditions**

1. Electrostatics: Poisson-Boltzmann Equation
   [#1] Positive bias **(V_in)**: 200 [mV]
   [#2] Negative bias **(V_out)**: 0 [mV]
   [#4] Surface charge density **(sc_mem)**: 0, -20 [mC/m^2^]
2. Transport of Diluted Species: Nernst-Planck Equation
   [#1~#4] No flux: $-n\cdot N=0$
3. Laminar Flow: Navier-Stokes Equation
   [#1~#2] Outlet: 0 [Pa]
   [#3~#4] Wall: u = 0
